# Investigation and analysis of sleep and mental health status among MEFCs

**DOI:** 10.3389/fpsyt.2025.1458291

**Published:** 2025-01-28

**Authors:** Kangying Yu, Shaozheng Song, Liu Wu, Zhe Chen

**Affiliations:** ^1^ School of Nursing, Taihu University of Wuxi, Wuxi, China; ^2^ Department of Psychiatry, The Affiliated Mental Health Center of Jiangnan University, Wuxi, China

**Keywords:** MEFC, sleep status, mental health, investigation, elderly people

## Abstract

**Objective:**

To investigate the sleep status and mental health of migrants elderly who followed their children (MEFC) and analyze the influencing factors.

**Methods:**

A total of 583 MEFCs were surveyed using a general demographic questionnaire, the Pittsburgh Sleep Quality Index (PSQI) scale, and the Symptom Checklist 90 (SCL90) scale.

**Results:**

The mean PSQI score for MEFCs was 6.98 ± 0.17, and the average SCL90 score was 64.06 ± 2.03. Multiple linear regression analysis indicated that health status and adaptation to the migration destination were associated with PSQI scores (P < 0.05). Health status, adaptation to the migration destination, and family harmony were associated with SCL90 scores (P < 0.05). The association coefficient between the total PSQI and SCL90 scores was r=0.462 (P < 0.05).

**Conclusion:**

The sleep and mental health of MEFCs were at a normal level. Health status and adaptation influenced sleep status, while health status, adaptation, and family harmony impacted mental health. However, the association between mental health and sleep status was weak.

## 1 Introduction

Population aging is a problem that many countries and regions are facing (1). The World Health Organization predicts that by 2030, approximately one sixth of the global population will be over the age of 60 (2). By 2050, this proportion will rise to about one-fifth, and by then, the number of elderly people will reach 2.1 billion (3, 4). China is also facing the problem of an aging population (5). According to the Seventh National Census Bulletin of China (6), the population aged 60 and above has reached 260 million, accounting for 18.7% of the total population. The 2022 World Population Prospects report predicts that the elderly population aged 60 and above in China will significantly increase in the first half of the 21st century (7). It is expected that this population will reach its peak around 2050, with a total of approximately 580 million people. By 2050, 26.9% of China’s population will be over 65 years old (8–10). In recent years, the rapid growth of the Chinese economy has triggered changes in the social structure (11). The urbanization process in China has rapidly improved, with 49.68% in 2010 and 63.89% in 2020 (12, 13). More than 200 million rural people have migrated to cities and temporarily reside in there to obtain better employment opportunities (14). With the large-scale migration of people, China’s traditional family structure(all family members and relatives reside in the same area) is being disrupted. Many adults migrate for employment, leaving their original families behind, thus giving rise to special groups such as left-behind children (15), elders left behind (16), empty-nesters (17), solitary elders (18), and migrants elderly following their children (19). This article focuses on MEFCs(migrant elderly who relocate to follow their children).According to the Seventh National Census conducted by the National Bureau of Statistics, China has 264 million elderly individuals aged 60 and above, of which 13.72 million are part of the mobile population, with MEFCs accounting for 43% of this group (6). This study adopts the definition proposed by Fanlei Kong, they are elderly individuals who relocate to other regions to care for their grandchildren or support their children. The age of elderly individuals should be over 55 years old at least (20). Research on this group is limited internationally, with most studies focusing on China (21–23). This disparity may arise because migration patterns for elderly individuals differ between China and other countries. In Western countries, elderly individuals typically migrate voluntarily to access better retirement services, while in China, elderly individuals migrate mainly for family obligations, often not entirely by voluntary choice. They may have unique physical or mental difficulties (23). This study aims to examine sleep quality as a survival indicator and explore its relationship with mental health. By focusing on the sleep and mental health of MEFCs, this study seeks to understand their living conditions and provide a theoretical basis for future improvements in their quality of life.

Sleep is the longest activity that occupies 24 hours a day, and it is an important indicator of quality of life. Compared to young people and children, elderly people are more likely to experience sleep disorders (24, 25). In summary, common sleep disorders among elderly people include insomnia, early awakening, frequent nighttime awakenings, obstructive sleep apnea, restless leg syndrome, and so on (14, 26–28). But not all elderly people will experience sleep disorders, and the frequency of sleep disorders varies in different regions. For example, the incidence of insomnia among the elderly population ranges from 6-60% in different regions and it is not affected by rural or urban areas, developed or underdeveloped countries (29). Hublin (30) and Ursin’s (31) survey found that the prevalence of sleep disorders in Finland and Norway is 20.4% and 20% respectively. The prevalence of sleep disorders among Asian immigrants in the United States is 18.2% (32).The prevalence of sleep disorders among elderly people in China is 46% (33).There are few studies specifically studying the sleep status of MEFCs, but there is evidence to suggest that their sleep status may be better than that of elderly people living alone (34).

The World Health Organization defines mental health as: mental health is a good adaptive state, in which individuals are in a state of self harmony, actively interact with the environment, and can give full play to their mental potential (35). mental health includes emotional and social role taking components, as well as social skills and cognitive functions that affect participation in basic tasks and social roles (36). The elderly are prone to mental health problems due to withdrawal from social work, physical function decline and other reasons (37), In the past, there were many reports on the mental health problems of the elderly (38–41). According to a survey by the National Health Commission, 30.3% of older people in urban areas have mental health problems, while only 26.8% of older people in rural areas have mental health problems (42). According to China’s national mental health development report (released in 2021), depression is one of the common mental symptoms of the elderly in China. About one fifth (19.05%) of older people are in a mild depression state, and nearly one tenth (12.17%) of older people have moderate to high depression (43). There are also many reports on the mental health of the migrant elderly (44), most of which focus on depression and anxiety (45), loneliness (46), social adaptation and integration (47), and social support (48), while there are few studies on other aspects.

Regarding the influencing factors of sleep, some studies suggest that a decrease in melatonin secretion in the elderly can lead to more shallow sleep and less deep sleep in their sleep structure, making them prone to problems such as going to bed early, waking up early, and waking up frequently at night (24). Bad physical conditions, such as hypertension, diabetes, cardiovascular disease, depression, stress, cognitive impairment are also related to sleep disorders. Demographic characteristics and lifestyle factors may have an impact on the sleep quality of older people. For example, factors such as female gender, low education, divorce and widowhood, living alone, insufficient fruit intake, drinking tea, drinking alcohol, caffeine intake, use of certain drugs, falls, pain, and lack of physical activity are associated with poor sleep quality (49–51). Many studies have discussed the factors that affect mental health. One study summarized four dimensions that affect the mental health of the elderly: People’s own conditions (health status, people’s attitude, behavior, etc.), the impact of living places (public open space and residence, etc.), the impact of social, cultural and economic environment in life, and the impact of natural or man-made major disasters. As for the research on the relationship between sleep and mental health, previous studies believed that sleep and mental health may have a certain correlation (52, 53). However, its causal relationship has not yet been clear. We noticed that when studying the relationship between sleep and mental health of the elderly, most studies focused on the relationship between sleep status and depression, anxiety, loneliness (49, 52). Research on the relationship between other aspects of mental health and sleep is limited, and studies exploring the connection between sleep and the mental health of MEFCs are scarce.

Research on MEFCs first appeared in 2010 (54). Many studies have explored the problems of depression, anxiety, loneliness and social support of MEFCs (46, 55–57). However, there are few studies on the sleep problems of MEFCs (58). There are also few studies on the relationship between sleep and mental health of MEFCs (59). Our research will fill this gap. We investigated the sleep and mental health status of MEFCs, and analyzed its influencing factors and correlation, which will provide a theoretical basis for improving the quality of life of MEFCs.

## 2 Materials and methods

### 2.1 Research design

This was a cross-sectional survey conducted between June 20 and September 20, 2022. The study employed a convenient sampling method, conducting surveys in Shanghai, Jiangsu, Zhejiang, and Fujian provinces, targeting eligible migrant elderly individuals with questionnaires, including a general demographic questionnaire, the Pittsburgh Sleep Quality Index (PSQI), and the Symptom Checklist 90 (SCL90). This study did not involve animal or human experiments; all surveys were conducted with participants’ consent, the ethics committee of the Wuxi Taihu university approved the study.

### 2.2 Participants

Inclusion Criteria: MEFC aged 55 years or older who have left their original place of residence to live with their children settled elsewhere for more than six months. Exclusion Criteria: Individuals aged below 55 years, residents living locally, or those who have migrated for less than six months. Sample Size: A total of 626 questionnaires were distributed, with 583 valid questionnaires retrieved after excluding invalid responses, yielding a valid response rate of 93%.

### 2.3 Survey instrument

Relevant surveys were conducted using a general demographic questionnaire, the PSQI, and the SCL90 scales. The general demographic questionnaire included variables such as age, gender, educational background, health status, adaptation to the migration destination, family harmony level, income level, community facilities, and social activity participation.

The PSQI scale was used to evaluate participants’ sleep quality over the past month. The scale was developed by Buysse (60). It comprises 19 self-rated items and 5 observer-rated items, with 18 items forming 7 dimensions. Each dimension is scored on a scale of 0–3, and the total score ranges from 0 to 21, where higher scores indicate poorer sleep quality. A score of 0–5 indicates very good sleep quality, 6–10 indicates fair sleep quality, 11–15 indicates average sleep quality, and 16–21 indicates very poor sleep quality, the Cronbach’s α of Chinese PSQI is 0.994 (61).

The SCL90 scale is effective at distinguishing individuals with mental symptoms (potentially at risk of or already experiencing mental disorders). It is suitable for identifying a group’s potential mental disorders, types, and severity. It can also be used to examine the presence of psychosomatic illnesses. According to the Chinese normative data results, an SCL90 total score above 160 or an average item score above 2 suggests that further evaluation is needed. A score exceeding 200 indicates significant mental problems warranting mental counseling, while a score above 250 suggests more severe issues requiring detailed medical assessment, potential mental therapy, or medication under medical supervision. Using the Chinese version of SCL-90, the cronbach’s α of the scale is 0.98, the cronbach’s α of each factor score ranges from 0.80 to 0.91, and the validity of the scale is 0.963 (62, 63).

### 2.4 Survey process

After receiving training on the background information, questionnaire content and social survey skills of the whole study, 50 college students became investigators. These students are all sophomores majoring in nursing. The respondents were searched in the community where the investigator was located. After a 25 minute face-to-face interview between the investigator and the respondent, a questionnaire survey was conducted after confirming that the respondent met the screening conditions. The paper questionnaire and electronic questionnaire are used. The electronic questionnaire is preferred. If the survey object is inconvenient, the paper questionnaire is used. The questionnaire was completed anonymously, and the paper questionnaire was collected on the spot, while the electronic questionnaire was collected after the participants submitted all of their questionnaires. 626 MEFCs were initially selected and a questionnaire survey was conducted. The unqualified questionnaires were deleted, and 583 valid questionnaires were finally collected.

### 2.5 Statistical analysis

Data entry and statistical analysis were conducted using SPSS 22.0 software. Categorical data were described using proportions and percentages; continuous data were described using means and standard deviations. Group comparisons were conducted using t-tests or analysis of variance (ANOVA). Pearson correlation analysis was used to assess relationships, and multiple linear regression was employed to identify factors influencing MEFCs’ sleep and mental health.

## 3 Result

### 3.1 Sleep quality and mental health status of MEFCs

**Table 1** presents the socio-demographic information of MEFCs. According to the data in **Table 1**, the study included 583 participants. The majority of participants were aged between 60 and 79 (62.4%), with 58.5% being female. Regarding education level, 26.1% of MEFCs participants had a high school education or above, while over half had a primary school education or below (50.9%). The monthly income level of MEFCs participants (48.8%) was less than 2000 yuan. Additionally, 52.2% of MEFCs participants suffered from chronic diseases.

**Table 1:** Basic characteristics of MEFCs and current sleep and mental health status (n=583).

Variables	N (%)	PSQI			SCL90		
		M (SD)	*F/t*	*P*	M (SD)	*F/t*	*P*
Gender							
Male	242 (41.5%)	6.91 ± 0.26	-0.345	0.73	64.36 ± 3.108	0.015	0.901
Female	341 (58.5)	7.03 ± 0.23			63.85 ± 2.701		
Age (year)							
55~59	193 (33.1%)	6.39 ± 0.26	3.469	0.032	54.64 ± 3.396		
60~79	364 (62.4%)	7.22 ± 0.23			68.46 ± 2.56		
>80	26 (4.4)	8.04 ± 0.94			72.08 ± 11.776	5.405	0.005
Educational background							
primary school education or below	297 (50.9)	7.04 ± 0.24	0.721	0.488	65.53 ± 2.87	0.61	0.543
Junior middle school	134 (22.9%)	7.21 ± 0.35			65.13 ± 4.335		
High school or above	152 (26.1%)	6.65 ± 0.32			60.28 ± 3.888		
Health status							
Unable to take care of oneself in daily life	13 (2.2%)	9.31 ± 1.49	10.025	<0.001	98.77 ± 15.312	19.792	<0.001
Capable of self-care, with chronic disease	322 (52.2%)	7.52 ± 0.24			73.28 ± 2.808		
very good and without diseases	248 (42.5)	6.16 ± 0.24			50.29 ± 2.764		
Differences between the place of migration and the original place of residence							
Not much difference	358 (61.4%)	6.55 ± 0.21	10.314	0.001	57.72 ± 2.406	15.878	<0.001
Significant difference	225 (38.5%)	7.67 ± 0.28			74.2 ± 3.544		
Whether to adapt to the place of relocation							
adapted	481 (82.5%)	6.55 ± 0.21	29.465	<0.001	89.67 ± 5.75	36.212	<0.001
Not adapted	102 (17.4%)	7.67 ± 0.28			58.52 ± 2.062		
Family harmony							
very harmonious	323 (35.4%)	6.48 ± 0.22	5.664	0.004	54.5 ± 2.605	14.879	<0.001
average	240 (41.1%)	8.48 ± 1.13			91.29 ± 10.924		
not harmonious	20 (3.4%)	7.49 ± 0.27			74.02 ± 3.193		
Monthly income							
<2000RMB	285 (48.8%)	7.37 ± 0.25	3.561	0.029	66.8 ± 3.168	1.201	0.302
2000~4 000RMB	220 (37.3%)	6.82 ± 0.28			62.86 ± 2.936		
>4 000RMB	78 (13.3%)	6.03 ± 0.4			57.49 ± 5.443		
Community facilities							
There are few leisure activities that elderly people can participate in.	123 (21%)	8.02 ± 0.37	5.789	0.003	74.08 ± 4.478	4.287	0.014
There are some leisure activities that elderly people can participate in.	364 (62.4%)	6.82 ± 0.21			63.02 ± 2.495		
There are many leisure activities that elderly people can participate in.	96 (16.4%)	6.25 ± 0.42			55.07 ± 5.396		

The sleep quality of MEFCs was assessed using the PSQI scale, the total PSQI score was 6.98 ± 0.17, which falls into the “fair sleep quality” category. These results suggest that the overall sleep quality of MEFCs are within normal ranges.

Their mental health was evaluated using the SCL90 scale. The total SCL90 score was 64.06± 2.04, indicating “good mental health.” These results suggest that the overall mental health status of MEFCs are within normal ranges.

### 3.2 Factors influencing the sleep quality and the mental health of MEFCs

We investigated the relationship between nine items and sleep status and mental health status:: Gender (male/female); Age (55~59, 60~79, >80); Educational background (primary school or below, junior middle school, high school or above); Health status (unable to take care of oneself, capable of self-care with chronic disease, very good and without diseases);Is there a significant difference between the social and cultural environment of your new location and your previous place of residence? (not much difference, significant difference); Adaptation to the migration environment (adapted, not adapted); Family harmony level (very harmonious, average, not harmonious); Income level (<2000 yuan, 2000~4000 yuan, >4000 yuan); and Availability of recreational activities for the elderly in the community (few, some, many).

Multiple linear regression analysis revealed that health status and adaptation to the migration environment were significantly associated with the PSQI total score (P < 0.05), identifying them as the primary factors influencing sleep quality.

Multiple linear regression analysis showed that health status, adaptation to the migration environment, and family harmony were significantly associated with the SCL90 total score (P < 0.05), identifying them as the primary factors influencing mental health. Details are presented in **Table 2**.

**Table 2:** multivariate analysis of sleep status scores and mental health status among MEFCs with different characteristics(n = 583).

Variables	PSQI					SCL90				
	*β*	*SE*	*Beta*	*t*	*P*	*β*	*SE*	*Beta*	*t*	*P*
(constant)	9.58	2.195		4.364	<0.001	84.72	25.485		3.324	0.001
Gender	0.273	0.343	0.033	0.795	0.427	2.969	3.984	0.03	0.745	0.456
Age	0.492	0.333	0.065	1.478	0.14	6.408	3.862	0.07	1.659	0.098
Educational background	0.116	0.121	0.045	0.956	0.34	1.068	1.406	0.035	0.759	0.448
Health status	-0.846	0.335	-0.11	-2.527	0.012	-16.452	3.885	-0.178	-4.234	<0.001
Differences between the place of migration and the original place of residence	0.197	0.379	0.023	0.521	0.603	2.894	4.402	0.029	0.657	0.511
Adaptation situation	-1.4	0.5	-0.13	-2.799	0.005	-17.633	5.809	-0.137	-3.036	0.003
Family harmony	0.204	0.176	0.048	1.157	0.248	6.165	2.043	0.122	3.017	0.003
Monthly income	-0.241	0.272	-0.041	-0.887	0.375	2.3	3.157	0.033	0.728	0.467
Community facilities	-0.451	0.287	-0.067	-1.572	0.116	-3.14	3.329	-0.039	-0.943	0.346

### 3.3 Correlation analysis between mental health and sleep quality

The correlation analysis was conducted on the mental health and sleep status of elderly people, and the results are as follows. The correlation coefficient between the total score of mental health status and sleep status is 0.462 (P<0.05), indicating a positive correlation between the mental health status and sleep status of migrant elderly people, but the correlation is not high. Details are presented in **Table 3**.

**Table 3:** Correlation analysis between mental health and sleep quality.

Variables	SOM	OC	IS	DEP	ANX	HOS	PHOB	PAR	PDY	OF	SCL90	A	B	C	D	E	F	G	PSQI
SOM	1																		
OC	0.765**	1																	
IS	0.686**	0.827**	1																
DEP	0.786**	0.858**	0.856**	1															
ANX	0.827**	0.846**	0.840**	0.875**	1														
HOS	0.676**	0.730**	0.804**	0.741**	0.781**	1													
PHOB	0.691**	0.774**	0.795**	0.800**	0.808**	0.700**	1												
PAR	0.670**	0.773**	0.868**	0.783**	0.815**	0.831**	0.727**	1											
PDY	0.737**	0.813**	0.871**	0.865**	0.866**	0.786**	0.801**	0.852**	1										
OF	0.798**	0.786**	0.754**	0.816**	0.843**	0.731**	0.750**	0.716**	0.782**	1									
SCL90	0.858**	0.911**	0.913**	0.939**	0.944**	0.846**	0.865**	0.873**	0.922**	0.879**	1								
A	0.347**	0.283**	0.244**	0.309**	0.299**	0.249**	0.248**	0.216**	0.239**	0.370**	0.322**	1							
B	0.334**	0.320**	0.269**	0.328**	0.337**	0.302**	0.267**	0.266**	0.230**	0.407**	0.335**	0.423**	1						
C	0.105**	0.126*	0.151**	0.134**	0.128**	0.127**	0.085*	0.136**	0.121**	0.204**	0.146**	0.191**	0.232**	1					
D	0.140**	0.085	0.126**	0.138**	0.126**	0.118**	0.085	0.113**	0.154**	0.160**	0.138**	0.167**	0.252**	0.635**	1				
E	0.561**	0.499**	0.455**	0.501**	0.534**	0.487**	0.456**	0.433**	0.438**	0.574**	0.551**	0.380**	0.489**	0.151**	0.171**	1			
F	0.270**	0.272**	0.333**	0.308**	0.301**	0.34**	0.359**	0.331**	0.342**	0.287**	0.338**	0.246**	0.175**	0.112**	0.162**	0.242**	1		
G	0.241**	0.210**	0.261**	0.264**	0.263**	0.266**	0.251**	0.254**	0.297**	0.273**	0.281**	0.360**	0.311**	0.055	0.038	0.504**	0.212**	1	
PSQI	0.425**	0.388**	0.399**	0.435**	0.443**	0.414**	0.376**	0.379**	0.385**	0.281**	0.462**	0.590**	0.703**	0.560**	0.594**	0.697**	0.474**	0.605**	1

**There was a significant correlation at the 0.01 level (bilateral).

*There was a significant correlation at the 0.05 level (bilateral).

SOM, somatization;OC,obsessive-compulsive disorder; IS, interpersonal sensitivity; DEP, depression; ANX, anxiety disorder; HOS, hostility; PHOB, phobic anxiety disorder; PAR, paranoid perception; PDY, psychosis; OF, other factors, SCL90,the total scares of SCL90 (62), A, Subjective sleep quality; B, Sleep latency; C, Sleep Continuity; D, Habitual sleep efficiency; E, Sleep disorder; F, Use of sleep medicine; G, Daytime dysfunction, PSQI, the total scares of PSQI.

## 4 Discussion

In this survey, we analyzed the sleep and mental health status of MEFCs from Jiangsu, Shanghai, Fujian, Guangdong and other provinces in China, and investigated and analyzed the influencing factors. Our study found that the overall sleep and mental health status of MEFCs were at a normal level, and their health status and community adaptation status would affect their sleep status; health status, community adaptation and family harmony will affect mental health status. Sleep and mental health showed a positive correlation, but the correlation was not significant.

The PSQI score for MEFCs in this survey was 6.98 ± 0.17, and the score falls within the normal range, indicating that MEFCs’ sleep quality are normal. In this study, 10.1% of MEFCs suffered from sleep disorders. This finding compares favorably with Wang’s survey results, which indicated that 18.3% of MEFCs had sleep disorders (58).

According to the survey on the mental health of MEFCs, our study found that the average score of mental health of MEFCs was 64.06 ± 0.17, which was within the normal range.These findings align with Fanlei Kong ‘s (64) study, which also reported normal mental health in MEFCs. However, they contrast with Xiaona Yu ‘s (65) findings, which suggested poor mental health among MEFCs. She believes that among MEFCs, our study is inconsistent with Wang’s results. He believes that mental health has become one of the biggest challenges faced by MEFCs (44).

The survey results further indicated that health status and adaptation to the migration environment were independent factors influencing sleep quality, with statistically significant differences (P < 0.05).This result is consistent with previous results. The influencing factors of sleep include health, mental, social support, aging, and so on (26). These influencing factors are also applicable to MEFCs. Poor health, poor social adaptation will cause sleep disorders in the elderly (51, 66).

The results showed that health status, adaptation to the migration environment, and family harmony level were independent factors affecting mental health, with statistically significant differences (P < 0.05).This result is consistent with previous studies, mental health is related to many factors, and many studies have explored the influence of various factors on mental health. Some studies believe that people, places, processes and living environments may affect mental health (67). Previous studies have also shown that social support (68), health status (69), family harmony (42), and social adaptation (46, 70) can affect mental health.

Correlation analysis between sleep quality and mental health showed a positive but weak correlation (r =0.462), indicating a minimal relationship between the two. This result is consistent with previous studies (71). Most of the previous studies explored the relationship between depression and anxiety and sleep. People with depression and anxiety are more likely to have sleep disorders, and sleep disorders will also be reduced when the symptoms of depression and anxiety improve. This study confirms this view (52, 72).

This study innovatively investigated the living conditions of MEFCs from the perspective of sleep and mental health. In previous studies, most studies focused on the mental conditions of the elderly, such as anxiety, depression, loneliness (64, 65, 73–79). However, there are few studies on the sleep status of MEFCs (58), and there is little literature on the relationship between sleep and mental health status of MEFCs (64). Our research will fill the gap in this field. This research will help us more comprehensively understand the living status of the MEFCs, and provide a theoretical basis for improving the quality of life of MEFCs.

This study has several limitations. First, the cross-sectional data used cannot effectively establish causal relationships. Second, mental health includes various aspects, such as anxiety and depression, which were not discussed in this study but should be considered in future research. Third, other factors, such as medication use, may influence elderly sleep quality. Additionally, health-related information could act as a confounding factor. Future research should explore the impact of such factors on the sleep and mental health of the elderly. Fourth, there is still a lack of sufficient data on the correlation between the dimensions of mental health and sleep status, and the accurate correlation needs to be more determined in the future. Lastly, although we tried to expand the number of questionnaires collected, the snowball sampling method used in this paper is also a limiting factor, which may cause insufficient data collection.

## Data Availability

The raw data supporting the conclusions of this article will be made available by the authors, without undue reservation.
